# Maternal-Fetal Physiology, Intrapartum Care, Postpartum Care: A Team-Based Learning Module for Normal Obstetrics

**DOI:** 10.15766/mep_2374-8265.10856

**Published:** 2019-11-22

**Authors:** Lindsey B. Sward, Sara G. Tariq

**Affiliations:** 1 Assistant Professor, Department of Obstetrics and Gynecology, University of Arkansas for Medical Sciences College of Medicine; 2 Professor, Department of Internal Medicine, University of Arkansas for Medical Sciences College of Medicine; 3 Assistant Dean of Undergraduate Clinical Education, University of Arkansas for Medical Sciences College of Medicine

**Keywords:** TBL, Team-Based Learning, Obstetrics, Gynecology, OB/GYN, Maternal-Fetal Physiology, Intrapartum Care, Postpartum Care, Postnatal Care

## Abstract

**Introduction:**

Team-based learning (TBL) is an active learning strategy used at the University of Arkansas for Medical Sciences in both the preclinical and clinical years of medical school. The Department of Obstetrics and Gynecology (OB/GYN) uses TBLs during a 6-week clinical clerkship. This TBL is the first in a series of six and was designed to teach the topic of normal obstetrics to third-year medical students.

**Methods:**

Prior to the TBL, students were provided with learning objectives and a list of advance preparation resources. These resources included a reading assignment from the student textbook, as well as optional online videos and optional online interactive quizzes. The students then came to class and completed an individual readiness assurance test (iRAT) and a group readiness assurance test (gRAT). The majority of in-class time was spent working through complex application exercises in the form of case vignettes. The TBLs were facilitated by a faculty member in the OB/GYN department.

**Results:**

Since its initiation in June 2018, 93 students have participated in this TBL activity. The mean score on the iRAT was 88.9%, and the mean score on the gRAT was 98.8%. Ninety-eight percent of students reported that they were satisfied with this learning activity.

**Discussion:**

This TBL was well received by students and unique in that it utilized a variety of types of advance preparation resources. With few other published OB/GYN TBLs available, we believe that this module could be a valuable resource for OB/GYN clerkships.

## Educational Objectives

By the end of this activity, learners will be able to:

1. Identify signs and symptoms associated with maternal physiologic changes of pregnancy, focusing on those related to the cardiovascular, respiratory, and hematologic systems.
2. Apply knowledge of the four stages of labor to clinical decision-making.
3. Interpret electronic fetal heart rate monitoring by describing fetal heart rate baseline, variability, and presence of decelerations.
4. Discuss normal anatomic and physiologic changes in the postpartum period (uterine involution, lochia, return of ovarian function, cardiovascular changes, breast changes).
5. Differentiate between postpartum blues, postpartum depression, and postpartum psychosis.

## Introduction

Obstetrics and gynecology (OB/GYN) is a diverse discipline that integrates preventive medicine, complex problem solving, and surgical intervention. Despite the complexity of the field, it is often relegated to a shorter clerkship length in medical school curricula.^1^ This makes planning of didactic sessions for students on the OB/GYN clerkship especially difficult, as these sessions must cover a significant amount of varied material over a short amount of time.

This team-based learning (TBL) activity was developed as the first in a series of six TBLs for a 6-week OB/GYN clerkship. It was designed to teach normal obstetrics—encompassing maternal and fetal physiology, intrapartum care, and postpartum care—to third-year medical students at the University of Arkansas for Medical Sciences (UAMS).

TBL is an active learning strategy that has been used in medical schools throughout the United States since 2001.^2^ TBL is characterized by three components—preparation in advance by the students, individual readiness assurance tests (iRATs) and group readiness assurance tests (gRATs), and the majority of in-class time spent focusing on application assignments.^2,3^ TBL has been shown to have a positive impact on the learning of medical students.^4^ Despite the noted benefits of TBL, there is a paucity of data on the use of TBL in OB/GYN clinical clerkships.^5^ A search of *MedEdPORTAL* revealed only six TBL activities specifically related to the field of OB/GYN.^6–11^

In comparing this TBL activity to the other six published in *MedEdPORTAL*, only one of them, “Post-Partum Depression: Diagnosis, Screening, and Treatment,” by Freerksen, Brooks, and Siddiqui,^8^ contains overlapping content. There are no published TBLs covering the topics of maternal and fetal physiology, basic intrapartum care, or comprehensive postpartum care. In contrast to the other published TBLs, this TBL activity utilizes a variety of different modes of advance preparation resources, whereas the other OB/GYN TBLs use textbook reading only.^6–11^ Finally, this TBL activity uniquely incorporates a significant amount of basic science into a clinical clerkship curriculum, with a focus on maternal and fetal physiology and maternal physiologic changes during the puerperium.

## Methods

### Curricular Context

Although the OB/GYN clerkship at UAMS has used TBL since 2007,^6,7^ a new TBL series was developed and instituted in June 2018. This TBL was the first in the new six-part series of TBLs given over a 6-week OB/GYN clerkship. Medical students at our institution were familiar with the TBL model of active learning as they had completed several TBLs during their preclinical years. The class size at UAMS at the time of the initiation of this TBL was 174 students. Third-year medical students rotated through their OB/GYN clerkship in groups ranging on average from 18 to 24 students.

In our OB/GYN clerkship, TBL in its entirety accounted for 20% of the student's grade. This module, as one of six TBLs, corresponded to approximately 3.3% of the final clerkship grade.

### Team Formation

At the beginning of each new clerkship, we randomly divided students into four different teams of five to seven students. These teams remained unchanged throughout the 6 weeks of the OB/GYN clerkship but were not consistent with teams in which students may previously have been placed in other courses.

### Description of Advance Preparation Resources

We sent the students their TBL learning objectives and a list of advance preparation resources 1 week prior to the TBL activity. The preparatory assignment for this TBL included a review of chapters in their textbook, *Obstetrics and Gynecology,* seventh edition.^12^ Students were asked to read chapter 5 (“Maternal-Fetal Physiology”), chapter 8 (“Intrapartum Care”), and chapter 11 (“Postpartum Care”) in their entireties. They were also asked to read only the portion of chapter 9 (“Abnormal Labor and Intrapartum Fetal Surveillance”) regarding intrapartum fetal surveillance. All items tested on the readiness assurance tests could be answered using the textbook reading alone.

We also encouraged the students to watch optional online videos produced by the Association of Professors of Gynecology and Obstetrics (APGO)^13^ that corresponded to the aforementioned chapters. The videos, 5- to 10-minute educational videos on various OB/GYN topics, were entitled Medical Student Educational Objectives and could be accessed on YouTube. For this TBL, the videos that corresponded to the reading material were topics 8 (Maternal-Fetal Physiology), 11 (Intrapartum Care), 13 (Postpartum Care), 14 (Lactation), 26 (Intrapartum Fetal Surveillance), and 29 (Anxiety and Depression).

We also recommend that the students complete the optional corresponding APGO online quizzes, called uWISE objectives.^14^ These quizzes were part of an online question bank to which our department had purchased a subscription. The question bank could be accessed, free of charge, by any student on our clerkship. The recommended uWISE objectives for this TBL were 8 (Maternal-Fetal Physiology), 11 (Intrapartum Care), 13 (Postpartum Care), 14 (Lactation), 26 (Intrapartum Fetal Surveillance), and 29 (Anxiety and Depression). Although completion of the uWISE objectives was not required for the TBL, we did award students a small completion grade at the end of the OB/GYN clerkship for completing all recommended uWISE objectives.

### Description of the Readiness Assurance Process

Students began the TBL by taking an iRAT (Appendix A). The iRAT was a 10-item multiple-choice quiz, given as a closed-book, closed-note quiz. We did not grade the iRAT prior to the students’ taking the gRAT. The students next broke into their teams to take the gRAT, which was the same 10-item multiple-choice quiz that they had been given for the iRAT (Appendix A). The TBL facilitator, who was a faculty member in the OB/GYN department and also a content expert, graded each gRAT upon its completion using a key (Appendix B). The facilitator gave immediate verbal feedback to the team about which items had been missed. Although we used a paper examination and key at our facility for financial reasons, we recognize that Immediate Feedback Assessment Technique cards^15^ could easily be adopted into this TBL activity.

Teams were allowed to use their textbooks or other materials to appeal questions. Questions were able to be appealed on the basis of poor or ambiguous wording or if the team believed that the answer was incongruent with information provided in the advance preparation materials. The teams were required to give appeals in writing for the faculty facilitator to review. Appeals could be written only by teams, not by individual students, and had to be turned into the faculty facilitator for review within 5 minutes of the grading of the gRAT.

After the gRAT was completed and any appeals reviewed, the TBL facilitator clarified difficult or poorly understood concepts with the students.

### Description of Team Application Activities

There were four application exercises in this TBL module (Appendix C), each designed according to the 4S principles of significant problem, same problem, specific choice, and simultaneous reporting.^2^ These exercises were structured as clinical vignettes with corresponding multiple-choice questions. Students worked in their groups to answer these complex clinical vignettes and were allowed to use a variety of resources, including textbooks and online components. Teams were asked to commit to an answer via simultaneous reporting.^3^ We conducted simultaneous reporting by having each team hold up a laminated card labeled A, B, C, D, or E corresponding to its answer. The facilitator then discussed the questions and answers with the groups.

### Facilitation Schema

This TBL was designed as a single, 90-minute educational session. The following is a suggested facilitation time line:

• Welcome and learning objectives (2-3 minutes).
• iRAT (15 minutes).
• gRAT (10 minutes).
• Appeals, clarification of difficult concepts (15 minutes).
• Application exercises (45 minutes).

## Results

Third-year medical students beginning in June 2018 participated in this TBL activity, with a total of 93 participants. The mean iRAT score on this TBL activity was 88.9%, with a minimum score of 50% and a maximum score of 100%. The median IRAT was 90%. The mean gRAT score was 98.8%, with all groups scoring either 90% or 100%.

Application exercises were not scored but were for discussion only. Although application exercise number 4 was almost universally agreed upon by all groups, there was more discussion regarding application exercise numbers 1-3, with groups typically being divided between two answer choices.

After participating in the TBL, students were sent an optional anonymous survey by email. The survey polled students on resources used and amount of time spent studying. The survey's response rate was 66% (61 out of 93 students). When students were asked which resources—reading, APGO videos, and uWISE objectives—they used to prepare for the TBL, 97% said they used the APGO videos, 74% said they used the uWISE objectives, and 67% said they used some or all of the reading. When asked which resources they found helpful for the TBL (with the ability to choose more than one resource type on the survey), 47% said they found the reading helpful, 75% said they found the APGO videos helpful, and 33% said they found the uWISE objectives helpful. The students polled spent 4 hours on average preparing for the TBL.

Students formally evaluated this TBL activity using a standard evaluation form utilized for all lectures and educational activities in the UAMS College of Medicine curriculum. The evaluation was voluntary and was scored on a 5-point Likert scale (1 = *strongly disagree*, 2 = *disagree*, 3 = *neither agree nor disagree*, 4 = *agree*, 5 = *strongly agree*). On an evaluation item stating, “Instructor provided learning material that was appropriate and well-organized, defined the learning objectives well, emphasized the stated objectives, presented lectures in an organized fashion, summarized major points, and illustrated relationships among topics,” 98% (50 out of 51 students) completing evaluations rated this TBL either a 4 or a 5. Of those, 94% (48 out of 51 students) rated it a 5. There were a total of 19 open-ended student responses from the evaluations, all of which were positive in nature. Select student comments regarding the TBL activity included: “So organized, energizing, and helpful”; “The topics were covered very well, and I left feeling much more confident in being able to know the topics when seen in clinicals”; and “The TBL format encourages student involvement. I think most didactic sessions for the M3 year could improve by taking tips from [this instructor].”

## Discussion

The UAMS OB/GYN clerkship has used TBL as a teaching method for more than a decade,^6,7^ but previously, little attention had been paid to the organization or quality of the TBL content. In the past, each TBL faculty facilitator wrote his or her own readiness assurance tests and application exercises, and TBLs were often not structured around a framework of learning objectives. This resulted in TBL activities that covered a wide variety of often-unrelated topics and did not consistently meet the strict definition of TBL. With lessons learned from past experience in mind, we desired to create a series of structured TBL activities that covered naturally interrelated content using truly active methods. A large amount of material must be taught in the OB/GYN clerkship in a span of time that is often shorter than that allotted to other clerkships^1^—because of this, we found it imperative to prioritize content we felt to be foundational and then to organize it in a logical manner.

Besides the challenge of covering large amounts of material, we also faced the challenge of incorporating new faculty members into TBL sessions. The majority of these faculty members had not been trained in TBL techniques. As a part of overcoming this barrier, we worked to create robust multiple-choice questions and application exercises that met the criteria of a true TBL session as outlined in the literature.^2,3^ This eased faculty burden in that these materials facilitated active learning, requiring that faculty learn only how to best proctor a TBL session.

This TBL activity utilized active learning strategies to teach basic knowledge of normal obstetrics to third-year medical students. We feel that this TBL could easily be adapted for use by other OB/GYN educators as an introductory didactic session for obstetrics in both traditional and longitudinal clerkship settings. The TBL was well liked by medical students, with 98% of them either agreeing or strongly agreeing that the TBL was an effective learning event. Faculty facilitators also voiced their satisfaction with this TBL as a learning activity. As there are few published OB/GYN TBL activities,^6–11^ our TBL could be a noteworthy contribution to an active learning–centered curriculum for OB/GYN clerkships.

We believe that this TBL is unique in that it uses a variety of different types of advance preparation resources. Traditional textbook reading has been combined with optional online educational videos and optional online quizzes. The videos and online quizzes allow students with different learning styles access to materials in their preferred learning style.^16^ The uWISE online quizzes are also interactive, and interactive learning tools such as online question banks have been associated with better medical student performance.^17^ We curated a list of several different types of advance preparation resources, all designed to teach the student the same material but in different ways. Based on the students who responded to a poll sent after the TBL's completion, a large majority of them utilized the APGO videos and the uWISE objectives in preparing for the TBL. In terms of which resources students found helpful in preparing for the TBL, 75% responded that the APGO videos were helpful, which was a higher percentage than those reporting the textbook reading to be helpful. Although the uWISE quizzes were not as highly regarded in terms of helpfulness as the APGO videos, one-third of students still found them helpful.

A current limitation of this resource is that we do not know if it will lead to improved student performance on OB/GYN standardized examinations. As this particular TBL series was implemented only this academic year, there are no current trends in grades on the National Board of Medical Examiners OB/GYN shelf examination that would lead us to believe the material is being mastered more effectively. We will continue to look for these trends, however. We will also begin to look for possible improvements in OB/GYN content on the United States Medical Licensing Examination Step 2 CK (Clinical Knowledge) exam.

Overall, this TBL activity was well organized, was well liked by students and faculty facilitators, and incorporated several different learning activities to teach a complex topic. As published TBL modules in OB/GYN are few, with no published resources on the majority of the material covered here, this TBL could serve as a valuable resource for other OB/GYN clerkships looking to adopt TBL as an active learning strategy.

## Appendices

A. RAT Student Version.docx
B. RAT Instructor Version.docx
C. Application Exercise Instructor Guide.docx All appendices are peer reviewed as integral parts of the Original Publication.
