# Supplementary material for: Maternal-Fetal Physiology, Intrapartum Care, Postpartum Care: A Team-Based Learning Module for Normal Obstetrics
Source: MedEdPORTAL. 2019 Nov 22;15:10856. doi: 10.15766/mep_2374-8265.10856 (PMC7050659; doi:10.15766/mep_2374-8265.10856)
Supplement: Supplementary file 1 — A. RAT Student Version.docx B. RAT Instructor Version.docx C. Application Exercise Instructor Guide.docx [file mep-15-10856-s001.zip › A. RAT Student Version.docx]

**ATTENTION, STUDENTS:** If you are accessing this material **BEFORE** it is used in your course, please do **NOT** read this document prior to the class session. An answer key is included in this module, which is designed to lead you through a learning experience that reinforces your knowledge of the content. Early review or dissemination of this material to others will diminish the learning opportunity and be considered academic misconduct.

TBL 1 – Normal Obstetrics

IRAT

Ms. Jones is a 28 yo G1 at 32 wks gestation who presents for a routine prenatal care visit. Today, she complains of shortness of breath, dizziness, and bilateral lower extremity swelling. BP 110/64. Pulse 99. Respirations 15. Pox 98% on room air. Physical exam reveals lungs that are clear to auscultation bilaterally, a 3/6 systolic murmur best heard at the left upper sternal border, and bilateral lower extremities with 1-2+ edema. The fetal heart rate is normal at 157.

1. Which of the following is the most likely cause of her shortness of breath?
2. Pulmonary embolism
3. Asthma exacerbation
4. Upper respiratory infection
5. Physiologic increase in tidal volume
6. What is the normal blood volume expansion during pregnancy?
7. 10%
8. 20%
9. 40%
10. 60%

Ms. Jones continues to see you for the remainder of her pregnancy and is now at 38 wks gestation. She presents today with elevated blood pressures and complaints of headache. You diagnose her with preeclampsia. You send her to labor & delivery for admission. On electronic fetal heart rate monitoring, you note late decelerations.

1. What would be the physiologic explanation for the late decelerations?
2. Umbilical cord compression
3. Uteroplacental insufficiency
4. Profound fetal anemia
5. Fetal vagal response

After further observation and resuscitative efforts, the late decelerations resolve. You proceed with delivery by induction of labor. Her cervix is dilated 2 cm, 50% effaced, with fetus at -3 station.

1. What is the definition of effacement?
2. Shortening of the cervical canal
3. The level of the fetal presenting part in relation to the ischial spines
4. Percentage of the fetal presenting part palpated in the vaginal canal
5. Width of the cervical opening
6. Which of these best describes Ms. Jones’s current stage of labor?
7. Second stage
8. Third stage
9. Active phase of the first stage
10. Latent phase of the first stage

Ms. Jones goes on to have a vaginal delivery of a healthy 3100 g male neonate. She has an uncomplicated postpartum course. Two weeks out from delivery, she calls your office complaining of a whitish vaginal discharge. She denies vaginal irritation or odor.

1. What is the most likely cause of her vaginal discharge?
2. Bacterial vaginosis
3. Candida infection
4. Lochia alba
5. Lochia rubra

Six weeks after delivery, you see Ms. Jones for a routine postpartum visit. She is exclusively breastfeeding her infant. She does complain of a painful lump in her left breast that has been present for about a week. She is afebrile and well-appearing. On exam, you note a localized area of swelling on the left breast that is mildly tender to palpation.

1. In women who exclusively breastfeed, what is the average time to resumption of ovulation?
2. 45 days
3. 90 days
4. 60 days
5. 180 days
6. What is the most likely diagnosis for Ms. Jones’s breast mass?
7. Engorgement
8. Galactocele
9. Carcinoma
10. Mastitis

Ms. Jones goes on to tell you that she has been very tearful over the past 6 weeks. She feels that this is interfering with the care of her newborn.

1. What is the most appropriate treatment for Ms. Jones at this time?
2. Reassurance & support
3. Admission to an inpatient psychiatric facility
4. Benzodiazepines
5. SSRIs
6. Which of the following is true regarding postpartum depression?
7. It is usually self-limiting
8. It is more common in older mothers
9. It is more common in women with pre-existing bipolar disorder or schizophrenia
10. Personal or family history of depression is a strong predictor
