# Supplementary material for: Maternal-Fetal Physiology, Intrapartum Care, Postpartum Care: A Team-Based Learning Module for Normal Obstetrics
Source: MedEdPORTAL. 2019 Nov 22;15:10856. doi: 10.15766/mep_2374-8265.10856 (PMC7050659; doi:10.15766/mep_2374-8265.10856)
Supplement: Supplementary file 1 — A. RAT Student Version.docx B. RAT Instructor Version.docx C. Application Exercise Instructor Guide.docx [file mep-15-10856-s001.zip › B. RAT Instructor Version.docx]

**ATTENTION, STUDENTS:** If you are accessing this material **BEFORE** it is used in your course, please do **NOT** read this document prior to the class session. An answer key is included in this module, which is designed to lead you through a learning experience that reinforces your knowledge of the content. Early review or dissemination of this material to others will diminish the learning opportunity and be considered academic misconduct.

TBL 1 – Normal Obstetrics

IRAT

Ms. Jones is a 28 yo G1 at 32 wks gestation who presents for a routine prenatal care visit. Today, she complains of shortness of breath, dizziness, and bilateral lower extremity swelling. BP 110/64. Pulse 99. Respirations 15. Pox 98% on room air. Physical exam reveals lungs that are clear to auscultation bilaterally, a 3/6 systolic murmur best heard at the left upper sternal border, and bilateral lower extremities with 1-2+ edema. The fetal heart rate is normal at 157.

1. Which of the following is the most likely cause of her shortness of breath?
2. Pulmonary embolism
3. Asthma exacerbation
4. Upper respiratory infection
5. Physiologic increase in tidal volume

*Progesterone causes increased central chemoreceptor sensitivity to CO2, which results in increased tidal volume, increased minute ventilation, and a reduction in arterial pCO2. Dyspnea of pregnancy is believed to be a response to low arterial CO2 caused by the increase in minute ventilation.*

Obstetrics and Gynecology, 7^th^ ed., page 49

**6% of students missed this question on the IRAT, and all of these students answered “A”.**

1. What is the normal blood volume expansion during pregnancy?
2. 10%
3. 20%
4. 40%
5. 60%

*Circulating blood volume begins increasing by 6-8 weeks of gestation and reaches a peak of 45% by 32 weeks gestation.*

Obstetrics and Gynecology, 7^th^ ed., page 47

**8% of students missed this question on the IRAT. Of those 8%, 2/3’s answered “B” and 1/3 answered “A”.**

Ms. Jones continues to see you for the remainder of her pregnancy and is now at 38 wks gestation. She presents today with elevated blood pressures and complaints of headache. You diagnose her with preeclampsia. You send her to labor & delivery for admission. On electronic fetal heart rate monitoring, you note late decelerations.

1. What would be the physiologic explanation for the late decelerations?
2. Umbilical cord compression
3. Uteroplacental insufficiency
4. Profound fetal anemia
5. Fetal vagal response

*Late decelerations are associated with uteroplacental insufficiency due to either decreased uterine perfusion of decreased placental function.*

Obstetrics and Gynecology, 7^th^ ed., page 117

**This question was missed by only 3% of students on the IRAT. Those who missed it answered “A” or “D”.**

After further observation and resuscitative efforts, the late decelerations resolve. You proceed with delivery by induction of labor. Her cervix is dilated 2 cm, 50% effaced, with fetus at -3 station.

1. What is the definition of effacement?
2. Shortening of the cervical canal
3. The level of the fetal presenting part in relation to the ischial spines
4. Percentage of the fetal presenting part palpated in the vaginal canal
5. Width of the cervical opening

*Effacement is the shortening of the cervical canal from a length of about 2 cm to a mere circular orifice with almost paper-thin edges.*

Obstetrics and Gynecology, 7^th^ ed., page 95

**Only 2% of students missed this question on the IRAT, choosing either “B” or “D”.**

1. Which of these best describes Ms. Jones’s current stage of labor?
2. Second stage
3. Third stage
4. Active phase of the first stage
5. Latent phase of the first stage

*The latent phase of the first stage of labor encompasses early cervical dilation. The active phase of the first stage of labor is a time of more rapid cervical dilation and usually begins at 4 cm.*

Obstetrics and Gynecology, 7^th^ ed., page 96

**10% of students missed this question on the IRAT. Of those 10%, all but one student chose “C”.**

Ms. Jones goes on to have a vaginal delivery of a healthy 3100 g male neonate. She has an uncomplicated postpartum course. Two weeks out from delivery, she calls your office complaining of a whitish vaginal discharge. She denies vaginal irritation or odor.

1. What is the most likely cause of her vaginal discharge?
2. Bacterial vaginosis
3. Candida infection
4. Lochia alba
5. Lochia rubra

*Lochia rubra is menses-like bleeding that occurs in the first several days postpartum. This is followed by lochia serosa, a lighter discharge with less blood. Lochia alba follows this and can persist for several weeks. It is a whitish discharge.*

Obstetrics and Gynecology, 7^th^ ed., pages 129-130

**3% of students missed this question, choosing either “B” or “D”.**

Six weeks after delivery, you see Ms. Jones for a routine postpartum visit. She is exclusively breastfeeding her infant. She does complain of a painful lump in her left breast that has been present for about a week. She is afebrile and well-appearing. On exam, you note a localized area of swelling on the left breast that is mildly tender to palpation.

1. In women who exclusively breastfeed, what is the average time to resumption of ovulation?
2. 45 days
3. 90 days
4. 60 days
5. 180 days

*The average time to return of ovulation is 45 days in non-lactating women and 180 days in lactating women.*

Obstetrics and Gynecology, 7^th^ ed., page 130

**This is the most missed question on the IRAT, answered incorrectly by 46% of students. Of those who missed this item, 47% chose “B”, 41% chose “A”, and 12% chose “C”. This was the only item missed by any team on the GRAT.**

1. What is the most likely diagnosis for Ms. Jones’s breast mass?
2. Engorgement
3. Galactocele
4. Carcinoma
5. Mastitis

*A galactocele, or plugged duct, may result in a localized swollen, tender area on the breast postpartum but would not cause fever or systemic symptoms like mastitis. Engorgement, in contrast to galactocele or mastitis, would be bilateral, generalized tenderness and swelling.*

Obstetrics and Gynecology, 7^th^ ed., pages 131-132

**14% of students missed this question, with half of those choosing “A” and the other half choosing “D”.**

Ms. Jones goes on to tell you that she has been very tearful over the past 6 weeks. She feels that this is interfering with the care of her newborn.

1. What is the most appropriate treatment for Ms. Jones at this time?
2. Reassurance & support
3. Admission to an inpatient psychiatric facility
4. Benzodiazepines
5. SSRIs

*Postpartum depression can last from several weeks to several months postpartum and is characterized by feelings of sadness, anxiety and despair that interfere with activities of daily living. Postpartum blues are milder and self-limiting; they typically only last for 1-2 weeks postpartum. Postpartum depression should be treated with mental health counseling and antidepressants.*

Obstetrics and Gynecology, 7^th^ ed., pages 134-135

**12% of students missed this question, with the vast majority of them choosing “A”.**

1. Which of the following is true regarding postpartum depression?
2. It is usually self-limiting
3. It is more common in older mothers
4. It is more common in women with pre-existing bipolar disorder or schizophrenia
5. Personal or family history of depression is a strong predictor

*Postpartum depression is not self-limiting like postpartum blues; symptoms tend to worsen over several weeks. It is more common in women with a personal or family history of depression, in younger women, and in women with lower socioecomonic status. Postpartum psychosis is more common in woment with pre-existing bipolar disorder or schizophrenia.*

Obstetrics and Gynecology, 7^th^ ed., page 134

**6% of students missed this question. Of those 6%, 1/3 chose “A”, 1/3 chose “B”, and 1/3 chose “C”.**
