# Supplementary material for: Maternal-Fetal Physiology, Intrapartum Care, Postpartum Care: A Team-Based Learning Module for Normal Obstetrics
Source: MedEdPORTAL. 2019 Nov 22;15:10856. doi: 10.15766/mep_2374-8265.10856 (PMC7050659; doi:10.15766/mep_2374-8265.10856)
Supplement: Supplementary file 1 — A. RAT Student Version.docx B. RAT Instructor Version.docx C. Application Exercise Instructor Guide.docx [file mep-15-10856-s001.zip › C. Application Exercise Instructor Guide.docx]

**ATTENTION, STUDENTS:** If you are accessing this material **BEFORE** it is used in your course, please do **NOT** read this document prior to the class session. An answer key is included in this module, which is designed to lead you through a learning experience that reinforces your knowledge of the content. Early review or dissemination of this material to others will diminish the learning opportunity and be considered academic misconduct.

TBL 1 – Normal Obstetrics

Application Exercises

1. A 32 year-old G1 presents to you for a new prenatal visit at 12w0d by LMP. She reports almost constant nausea with vomiting multiple times per day for the past 4 weeks. This is affecting her daily life. She reports a pre-pregnancy weight of 145 lbs. The patient also reports urinary frequency and an increase in whitish vaginal discharge. On exam today, her weight is 135 lbs. Her blood pressure is 105/60, pulse 90. A urine dipstick is performed that is negative for ketones, nitrites, and leukocyte esterase. Urine dipstick is positive for small amount WBC and 2+ glucosuria. Wet prep of vaginal discharge is performed showing increased number of WBC per high-powered field. Ultrasound is performed, revealing an intrauterine gestation with crown-rump length equal to 11w3d. What is the next most appropriate step in management?
2. Treatment of urinary tract infection
3. Admission to the hospital for IV fluids, anti-emetics
4. Prescription for azithromycin for chlamydia infection
5. Basic metabolic panel to rule out electrolyte abnormalities
6. Discussion regarding helpful diet & lifestyle changes and OTC medications

*This patient with severe nausea and vomiting has lost more than 5% of her pre-pregnancy weight, possibly meeting the diagnosis of hyperemesis gravidarum. However, based on her vital signs and urine dipstick, she is not currently dehydrated. The next most appropriate step in management is to rule out electrolyte abnormalities (D). This could help to decide between inpatient and outpatient management. It is also important to discuss helpful diet and lifestyle changes and OTC medications for nausea and vomiting (E), but this would be more appropriate once electrolyte abnormalities and the possible need for inpatient admission are ruled out. The urine dipstick is positive for white blood cells and glucosuria; it is important to discuss with the students that these things are likely normal findings in pregnancy. Similarly, a vaginal discharge with increased number of WBC per high-powered field is also likely normal luekorrhea of pregnancy.*

**Student groups tend to choose “B” or “D” on this exercise.**

1. A 36 year-old G1 presents to L&D at 39 weeks’ gestation with rupture of membranes at home two hours prior to admission. On arrival, rupture of membranes is confirmed, and her cervix is noted to be 2 cm dilated. Fetal monitoring reveals baseline heart rate of 140 with frequent accelerations and no decelerations. Four hours later, the patient complains of feeling pressure. You recheck her cervix and find that it has not changed. She is contracting every 5-7 minutes. The fetal heart rate tracing remains reassuring. What is the most appropriate management?
2. Continue expectant management, as the patient will likely delivery spontaneously
3. Administer oxytocin to augment her labor
4. Prepare for cesarean delivery
5. Allow the patient to be discharged home, as she is not in active labor
6. Begin prophylactic antibiotics for prolonged rupture of membranes

*This patient is in the latent phase of the first stage of labor and also has premature rupture of membranes. Over a period of four hours, she has not had change in her cervical dilation. Because of the rupture of membranes, it is important to expedite the delivery process as much as possible to avoid ascending infection, so answer (A) of continuing expectant management is not the best. However, there is no need to proceed to cesarean section as the patient has not yet entered active labor and should not be considered to have arrested dilation. Her fetal states is reassuring as well, so there is no need for a cesarean delivery at this time. The patient should not be discharged home, as she has had rupture of membranes and should be delivered in a timely fashion. Option (B) – administering oxytocin to augment labor – is the most appropriate management in this situation. There is no need for prophylactic antibiotics as the patient has not had prolonged rupture of membranes. It would be good to discuss with the students what constitutes prolonged rupture of membranes and in what circumstances prolonged rupture of membranes would necessitate antibiotic therapy.*

**Student groups tend to choose “A” or “B” on this activity. Rarely, a group will choose “E”.**

A 22 year-old multigravida delivered her third healthy child vaginally without complication. You feel that the patient is ready for discharge from the hospital. She is breastfeeding, as she has with all of her children. She would like to discuss contraceptive options with you prior to her discharge because she “does not want another baby anytime soon”. Upon further questioning, she does have a history of postpartum depression after a prior pregnancy.

1. Which of these contraceptive options would be best for this patient?
2. Reassure her that she does not need contraception, as she plans to exclusively breastfeed
3. Combined oral contraceptive pill, to begin immediately
4. Combined oral contraceptive pill, to begin 21 days (3 weeks) postpartum
5. Depot medroxyprogesterone acetate (DMPA), injection can be given prior to discharge
6. Progestin-only oral contraceptive pill, to begin immediately

*In this postpartum patient who is breastfeeding, the recommendation on combined hormonal contraception would be to wait until at least 30 days from delivery before beginning combination oral contraceptive pills. This is due to the theoretical risk of estrogen on breast milk volume and composition. While most breastfeeding women will not resume ovulation until an average of 180 days postpartum, this delay in ovulation depends upon exclusive breastfeeding, every 4-6 hours. This is not the most reliable contraceptive method for this patient. Progestin-only methods of contraception can be started immediately postpartum in the breastfeeding woman, making (D) or (E) acceptable choices. DMPA would be more reliable than a progestin-only pill; however, DMPA has been shown in some studies to increase the postpartum depression score. Some experts recommend avoiding DMPA in the immediate postpartum period. It would be prudent to discuss this with the patient, though, who might prefer the more reliable DMPA over progestin-only pills, even in the setting of a history of postpartum depression.*

**Student groups tend to choose “D” or “E” on this activity. Occasionally, a group will choose “A”.**

The same patient presents to your office for an urgent visit 5 days postpartum. She reports that her breasts are very tender and swollen. She has been trying to breastfeed, but she has also been formula-feeding at times because her baby has not been latching well. On exam, she is in no distress but appears tired. Her temp is 100.0, blood pressure 120/70, pulse 85. Bilateral breasts are diffusely firm and tender to palpation. She does have small fissures on bilateral nipples.

1. What is the next best step in management?
2. Prescription for dicloxacillin
3. Bilateral breast ultrasound to evaluate for abscess
4. Referral to a lactation consultant
5. Encouragement to continue breastfeeding at regular intervals

*The patient has bilateral firm, tender breasts, most consistent with engorgement, which can give rise to mild temperature elevations. She does not have a true fever, systemic symptoms, or a localized area of redness or tenderness of her breast, so it is unlikely that she has mastitis or a breast abscess. Therefore, (A) and (B) are incorrect. The patient should be encouraged to continue to breastfeed at regular intervals (D) to decrease engorgement, but the infant is not latching appropriately based on the given history also on the nipple fissures noted in the physical exam. Nipple fissures are usually the result of inappropriate breastfeeding technique. A referral to a lactation consultant (C) would be the next best step in management for this patient.*

**The majority of student groups choose “C” on this exercise, though rarely a group will choose “D”.**
